# Extracellular HMGB1 interacts with RAGE and promotes chemoresistance in acute leukemia cells

**DOI:** 10.1186/s12935-021-02387-9

**Published:** 2021-12-21

**Authors:** Weixin Lai, Xinyu Li, Qian Kong, Han Chen, Yunyao Li, Lu-Hong Xu, Jianpei Fang

**Affiliations:** 1 Guangdong Provincial Key Laboratory of Malignant Tumour Epigenetics and Gene Regulation, Guangzhou, People’s Republic of China; 2 Department of Paediatrics, Sun Yat-Sen Memorial Hospital of Sun Yat-Sen University, 107 Yanjiang West Road, Guangzhou, 510120 Guangdong People’s Republic of China; 3 Department of Pediatrics, The Third Affiliated Hospital of Sun Yat-Sen University, Guangzhou, People’s Republic of China

**Keywords:** Acute leukemia, HMGB1, RAGE, Autophagy, Apoptosis, Drug efflux protein, Drug-resistance

## Abstract

**Background:**

Nowadays, acute leukemia (AL) among children has favorable outcome, yet some of them get refractory or relapse mainly due to drug resistance. High-mobility group box 1 (HMGB1) has been proven to have a important role in drug resistance via upregulation of autophagy after chemotherapy treatment in acute leukemia. However, the mechanism how extracellular HMGB1 acts on AL cells and leads to chemoresistance remains elusive.

**Method:**

CCK8 was used to examine the toxicity of chemotherapeutic drug. Elisa was performed to detect the release of HMGB1. Western blot and mRFP-GFP-LC3 adenoviral particles as well as transmission electron microscopy were used to detect the autophagy flux. Western blot and flow cytometry were applied to evaluate the apoptosis. qPCR and western blot were conducted to detect the expression of drug efflux protein. Lentivirus infection was applied to knock down RAGE. In addition, T-ALL NOD/SCID mice xenograft model was used to observe the effect of inhibiting HMGB1/RAGE axis.

**Results:**

We found that extracellular HMGB1 do upregulate autophagy and in the meantime downregulate apoptosis, primarily through interaction with receptor for advanced glycation end products (RAGE). Suppression of RAGE by RNA interference alleviated the level of autophagy and enhanced apoptosis. What’s more, HMGB1/RAGE induced autophagy was associated with the activation of ERK1/2 and decreased phosphorylation of mammalian target of rapamycin (mTOR), while HMGB1/RAGE limited apoptosis in a Bcl-2-regulated way mediated by P53. On the other hand, we found that HMGB1/RAGE activated the NF-κB pathway and promoted the expression of P-glycation protein (P-gp) as well as multidrug resistance-associated protein (MRP), both are ATP-binding cassette transporters. In vivo experiment, we found that blocking HMGB1/RAGE axis do have a mild pathological condition and a better survival in T-ALL mice.

**Conclusion:**

HMGB1/RAGE have a important role in drug resistance after chemotherapy treatment, mainly by regulating autophagy and apoptosis as well as promoting the expression of drug efflux protein such as P-gp and MRP. HMGB1/RAGE might be a promising target to cure AL, especially for those met with relapse and refractory.

## Introduction

Acute leukemia, the most common cancer happened in children, is originated from the abnormal proliferation and differentiation of hematopoietic stem cell (HSC), which results in the disturbance and deterioration of the function of bone marrow [1]. Acute leukemia has two main subtypes: acute lymphoblastic leukemia (ALL) and acute myeloid leukemia (AML), classified by their affected lineage [2, 3]. Nowadays, with the advancement in supportive care, chemotherapy treatment and risk stratification, treatment of acute leukemia happened in children has gained great achievement, with an overall cure rate of more than 70%. Particularly, the 5-year survival rate of ALL is above 85% in developed countries. However, in China, the 5-year event-free survival rate of ALL is about 82%, and the cumulative risk of relapse was 24.5% at 10 years, which often confers worse prognosis [4, 5]. Refractory and relapse often arise from the acquisition of drug resistance. Furthermore, the dysregulation of autophagy and apoptosis, as well as the upregulated expression of ATP-binding cassette transporters (transporter-mediated drug efflux), contribute greatly to the occurrence of drug resistance [6].

Autophagy, as a conserved cellular recycling process, plays an important role in maintaining homeostasis and stress adaptation. When cells undergo hypoxia, stress, starvation, chemotherapy treatment, autophagy is upregulated to clear the damaged organelles, degrade cellular components to increase nutrient availability and reduce toxic wastes, which promotes the survival of cancer cells [7, 8]. While apoptosis, also known as programmed cell death, also acts as a defense mechanism to eliminate damaged cells or cells with harmful mutations. And the dysregulation and evasion of apoptosis is the common and important aspect in the development of cancers, which is considered as the hallmark of cancers [9, 10]. The role of autophagy and apoptosis in chemotherapy treatments is complicated and depends on the progression of tumor, drug properties and so on. It is said that they are double-edge sword in treating cancers [11–13]. To clarify the relationship between autophagy and apoptosis in different stages of disease is beneficial for us to cure leukemia more reasonably.

Currently, a growing body of literature pointed out the central role of high-mobility group protein 1 (HMGB1) in regulating autophagy and apoptosis, which contributes greatly to the development of chemotherapeutic resistance [14–19]. HMGB1 is a highly conserved nuclear protein that has been proven to be upregulated in various kinds of cancers. Studies demonstrated that HMGB1 was over-expressed in three leukemia cell lines (K562, HL60 and Jurkat) and was correlated to the stage of leukemia [20]. And it is reported that the expression of HMGB1 in primary and relapsed leukemia was much higher than healthy people and patients with complete remission [21]. HMGB1 mainly locates in nucleus in quiescent condition. However, in response to stress or chemotherapy treatments, HMGB1 can translocate into the cytoplasm and excrete into extracellular matrix via post-translational modification such as acetylation, poly ADP-ribosylation and so on. Study showed that poly (ADP-ribosylation) of HMGB1 facilitated its acetylation and promoted HMGB1 translocation-associated chemotherapy-induced autophagy in leukemia cells in which acetylation of HMGB1 is the most crucial process [22]. In different parts of cell, HMGB1 has different functions. In nucleus, HMGB1 acts as DNA chaperone, participating in DNA repairs and sustaining the stability of chromosomal. What’s more, it can modulate the transcription of some genes such as P53 and NF-κB. Meanwhile, Tang et al. proved that HMGB1 could promote the transcription of HSP27 and then enhanced autophagy through Pink1/Parkin pathway. In cytoplasm, HMGB1 dislocates Beclin1–Bcl2 and contributes to the formation of Beclin1/PI3K-III complex to induce autophagy, mainly via MAPK/ERK signaling pathway. What’s more, it was demonstrated that HMGB1 can promote autophagy by activating PI3K/AKT/mTORC1 pathway. Extracellular HMGB1 also has been found to upregulate autophagy by activating AMPK/ERK pathway and AMPK/mTOR signaling pathway. Besides, receptor for advanced glycation end products (RAGE) was involved in this process [20, 23–26].

RAGE is an evolutionarily single transmembrane member of the immunoglobulin superfamily. RAGE binds to an array of structurally diverse ligands, which include advanced glycation end products, S100/calgranulin family, extracellular HMGB1 and so on, acting as a pattern recognition receptor (PRR) and is expressed on cells of different origins performing different functions. RAGE is the first reported receptor of HMGB1 with high affinity [27–30]. Studies showed that RAGE played an indispensable role in HMGB1 induced autophagy. And a number of studies demonstrated that RAGE was involved in the proliferation, differentiation, metastasis of tumor cells. What’s more, RAGE can alleviate the ROS level and sustain autophagy as well as suppress apoptosis in pancreatic tumor cells [30–33]. However, the role of HMGB1/RAGE axis have not been studied in AL.

Transporter-mediated drug efflux, such as P-gp and MRP, also have great association with the occurrence of drug resistance. Both P-gp and MRP are belong to ATP-binding cassette transporters, encoded by ABCB1/ABCC1. And they are able to eject a variety of substrates from cytoplasm to extracellular matrix, including chemotherapeutic agents [34]. A great number of studies asserted that drug efflux proteins have a close relationship with drug resistance. And it is believed that anthracycline agents such as adriamycin (ADM), daunorubicin (DNR), are prone to induce drug resistance by upregulating the expression of ATP-binding cassette transporters [35–37]. Furthermore, evidences showed that extracellular HMGB1 could upregulate the expression of P-gp and MRP in gastric adenocarcinoma cells. In status epilepticus rat brains, researchers found that extracellular HMGB1 regulated P-gp expression via RAGE/NF-κB signaling pathway. On the other hand, a number of studies showed that NF-κB was the downstream of HMGB1 [38, 39]. However, it still remain unknown whether HMGB1/RAGE has a relationship with NF-κB and the upregulation of ATP-binding cassette transporters in AL.

We have previously demonstrated that ULK1/2-ATG13-FIP200 complex, one of the critical effectors of autophagosome formation, acting as upstream of HMGB1-Beclin1, played a critical role in autophagy induced by DNR. What’s more, we found that poly (ADP-ribosylation) of HMGB1 facilitated its acetylation and promoted HMGB1 translocation-associated chemotherapy-induced autophagy in leukemia cells. In this study, we show that extracellular HMGB1 interacts with RAGE to sustain AL cells survival by promoting autophagy and decreasing apoptosis. What’ more, HMGB1/RAGE enhanced the expression of P-gp and MRP via activating the NF-κB pathway. These findings suggest that HMGB1/RAGE promotes tumor cell survival after chemotherapy treatments. Meanwhile, we provides some evidences for the development of novel clinical approaches targeting the HMGB1/RAGE pathway.

## Materials and methods

### Subjects and cell culture

The human acute lymphoblastic leukemia cell line Jurkat and the human acute myeloid leukemia cell line HL-60 were purchased from the Type Culture Collection of the Chinese Academy of Sciences; Both Jurkat and HL-60 cells were cultured in RPMI-1640 (Gibco; Thermo Fisher Scientific, Inc.) with 10% fetal bovine serum (Gibco; Thermo Fisher Scientific, Inc.) in a humidified incubator with 5% CO_2_ and 95% air.

### Antibodies and reagents

Antibodies specific for GAPDH (catalogue no. 5172S), β-actin (catalogue no. 3700S), HMGB1 (catalogue no. 6893S), LC3 (catalogue no. 3868S), Bax (catalogue no. 5023S), Bcl 2 (catalogue no. 15071S), NF-κB p65 (catalogue no. 8242S), phosphorylated p65 (catalogue no. 3033S), erk (catalogue no. 9102S), phosphorylated erk (catalogue no. H9539), were obtained from Cell Signaling Technology. Antibodies specific for P62 (catalogue no. ab56416), RAGE (catalogue no. ab3611), P-gp (catalogue no. ab170904), Cleaved caspase 3 (catalogue no. ab32042), phosphorylated mTOR (catalogue no. ab137133), mTOR (catalogue no. ab134903), phosphorylated P53 (catalogue no. ab33889), P53 (catalogue no. ab32389), PUMA (catalogue no. ab33906) were obtained from Abcam. Antibodies specific for MRP (catalogue no. abs116285) was obtained from Absin Bioscience, Inc. Antibodies specific for PARP (catalogue no. A0942) was obtained from ABclonal Technology, Inc. Antibodies that mentioned above were diluted to 1:1000 in 5% BSA to detect the target protein. The secondary antibodies, including sheep anti-mouse IgG-HRP (catalogue no. RM3001), sheep anti-rabbit IgG-HRP (catalogue no. RM3002) were obtained from Beijing Ray Antibody Biotech. They were diluted to 1:5000 in TBST (1% Tween 20). Recombinant Human HMGB1/High mobility group protein B1 (catalogue no. abs04694) was obtained from Absin Bioscience, Inc. Adriamycin (ADM), FPS-ZM1 were purchased from MedChemExpress.

### Drug treatment

Both Jurkat and HL-60 cells were treated with different concentrations of adriamycin (ADM) (0, 0.05, 0.1, 0.2, 0.4, 0.8 µM) for 24 h to test the cell viability. What’s more, we pretreated cells with extracellular HMGB1 (0, 25, 50, 100 ng/mL) for 24 h and then they were treated with ADM (0.4 µM). In the subsequent experiments, both Jurkat and HL-60 cells were treated with FPS-ZM1, a small molecular inhibitor of RAGE, or transfected with lentivirus to knock down the expression of RAGE. Then we pretreated them with extracellular HMGB1 (50 ng/mL) for 24 h, and then cells were incubated with ADM (0.2 µM) for another 24 h. Subsequently, lentivirus was used to regain the expression of RAGE in order to assert the function of RAGE. To figure out the role of P53, ERK2/1, mTOR and NF-κB in HMGB1/RAGE downstream signals, we then added their small molecular inhibitor pifithrin-β, PD98059, Rapamycin and pyrrolidine dithiocarbamate ammonium (PDTC) into cells to inhibit those pathways with or without HMGB1. Then they were treated with ADM (0.4 µM) for another 24 h.

### Cell viability assay

Both Jurkat and HL-60 cells were seeded in 96-well plates at a density of 3 × 10^4^/mL, after treated with different concentration of chemotherapeutic drug for 24 h, 10 μL of the Cell Counting Kit-8 solution (Dojindo Molecular Technologies, Inc.) was added into wells and incubated for 2 h. Then the absorbance at 450 nm was measured in a microplate reader.

### Quantitative real-time PCR (RT-qPCR)

Total RNA was isolated from both Jurkat and HL-60 cells using RNA-Quick Purification Kit (Yishan Biotech Inc.) and then reverse-transcribed into cDNA. The sequences of primers used were as follows: GAPDH: forward, 5ʹ-ATGACTCTACCCACGGCAAG-3ʹ and reverse, 5ʹ-TACTCAGCACCAGCATCACC-3ʹ; for HMGB1: forward, 5ʹ-CTGTCCATTGGTGATGTTGC-3ʹ and reverse, 5ʹ-CTGATAGCCTGCTCCAGGTC-3ʹ; for RAGE: forward, 5ʹ-GAATCCTCCCCAATGGTTCA-3ʹ and reverse, 5ʹ-GCCCGACACCGGAAAGT-3ʹ; for ABCB1: forward, 5ʹ-TGATTGCATTTGGAGGACAA-3ʹ and reverse, 5ʹ-CCAGAAGGCCAGAGCATAAG-3ʹ; for ABCC1: forward, 5ʹ-GGTTTATAGTAGGATTTACACGTGGTTG-3ʹ and reverse, 5ʹ-AAGATAGTATCTTTGCCCAGACAGC-3ʹ. Reactions were carried out in a Roche Light Cycler 96 system (Roche Diagnostics GmbH, Mannheim, Germany) with a SYBR Premix ExTaq kit (Takara Bio Inc., Otsu, Shiga, Japan). Data were normalized to GAPDH expression and analyzed with the 2^−∆∆Ct^ method.

### Western blot analysis

The two cell lines were subjected to the aforementioned different ADM concentrations and other chemical agents, then they were collected and lysed with RIPA buffer solution (Beyotime Institute of Biotechnology). The protein concentration was determined by BCA method (Beyotime Institute of Biotechnology). Samples (25 µg) were separated via SDS-PAGE (10 or 12% gel) and transferred onto a polyvinylidene fluoride (PVDF) membrane (EMD Millipore). After blocking with 5% Bovine serum albumin for 1 h at room temperature, the membranes were incubated with primary antibodies, including HMGB1, RAGE, p62, LC3II/I, Parp, P53, phospho-P53, bcl2, bax, Puma, cleaved caspase 3, erk, phospho-erk, NF-kB/p65, phospho-NF-kB/p65, MRP, P-gp overnight at 4 °C. Secondary antibodies, including sheep anti-mouse IgG-HRP and sheep anti-rabbit IgG-HRP were applied at a 1:5000 dilution for 1 h at room temperature. β-actin and GAPDH were used as loading controls to detect the expression in whole protein. The target protein expressions were detected with an enhanced chemiluminescence reagent (EMD Millipore) using a G:BOX XT4 system (Syngene).

### HMGB1 Elisa kit

The HMGB1 Elisa kit was purchased from Elabscience Biotechnology Co, Ltd. Both Jurkat and HL-60 cells are treated with ADM (0, 0.05, 0.1, 0.2, 0.4, 0.8 µM) for 24 h, then the supernate was collected. We diluted the supernate at 1:2 and added into the wells. The supernate and the reaction mixture were incubated in a 37 °C incubator for 30 min. Then the absorbance at 450 nm was measured in a microplate reader and we calculated each wells’ content of HMGB1 according to the standard curve.

### Lentivirus infection

To mutate the acetylation site of HMGB1, Jurkat cells that had been transfected with shRNA-mutated acetylation site of HMGB1 was purchased and from OBiO Technology Corp., Ltd and was verified. To knock down RAGE, we chose three potential sequences, and lentiviruses were purchased from HanBio Technology (Shanghai, China). Experiments were performed in 12-well plates at a density of 3 × 10^5^ cells/well. In the present study, both Jurkat and HL-60 cells were divided into three groups, which consisted of the wild type, cells transformed with lentivirus that included Normal control and shRNA. 2 ug/mL puromycin (Beijing Solarbio Science & Technology Co., Ltd.) was applied to select cells that had been well transfected. Then we used qPCR and western blot to measure the expression of RAGE. Finally, we selected the best one that targeting RAGE and the shRNA sequence was as follow: forward, 5ʹ-GCCGGAAAUUGUGAAUCCUTT-3ʹ and reverse, 5ʹ-AGGUUCACAAUUUCCGGCTT-3ʹ. In the subsequent experiment, lentivirus was used to re-express RAGE, western blot was used to detect the expression of RAGE. Cells were cultured at 37 °C in a humidified incubator containing 5% CO_2_ for 3–5 generations and then used for subsequent experiments.

### Detection of autophagic flux

The mRFP-GFP-LC3 adenoviral particles were purchased from HanBio (Shanghai, China). Cells were infected with adenoviral particles; after infection, the cells were pretreated with rHMGB1 (50 ng/mL) for 24 h before chemical treatment or were distinctly cultured with ADM (0.4 µM) for another 24 h. Then cells was fixed with 4% paraformaldehyde for 20 min and was washed with PBS twice. Imaging was performed on CarlZeiss LSM710 and analysis was performed using ZEN 2009.

### Transmission electron microscopy

The cells were fixed with 2% paraformaldehyde and 2.5% glutaraldehyde in 0.1 mol/L phosphate buffer (pH 7.4) at 4 °C for 2 h, followed by 1% OsO4. After dehydration and Epon-812:100% acetone embedding at room temperature, thin sections (50–80 nm) were stained with uranyl acetate and lead citrate respectively at 4 °C for 15 min, and viewed under a HITACHI (HT7700) election microscope (FEI; Thermo Fisher Scientific, Inc).

### Flow cytometry

Both Jurkat and HL-60 cells that transfected with shRNA-NC and shRNA-RAGE were pre-treated with or without extracellular HMGB1 (50 ng/mL) for 24 h, then 0.8 uM ADM was added for another 24 h. Then leukemia cells were washed with PBS for 2 times and then stained with Annexin V APC-A (ThermoFisher Scientific, US) and APC-Cy7-A (ThermoFisher Scientific) before its detection by flow cytometry (FACSCalibur, BD Biosciences, San Diego, CA).

### Animal study

Jurkat cells (5 × 10^6^/mL) that stably transfected with shRNA-NC, shRNA-RAGE as well as shRNA-mutated acetylated site of HMGB1 was injected into NOD/SCID mouse via tail intravenous injection. There are seven mice in each group. Low dosage of ADM (1 mg/kg) was injected into mouse by intraperitoneal injection every other day. 25% weight loss or live for 4 weeks were set as the end point. Then the spleen was removed to make pathological section by using hematoxylin–eosin staining. Furthermore, Bone marrow sediment of mouse was removed and stained with BV421-CD3 before flow cytometry to detect the proportion of leukemia cells in bone marrow. Part of them was subjected to Transmission electron microscopy to detect the level of autophagy. This study was approved by the Ethics Committee of the Sun Yat-Sen Memorial Hospital of Sun Yat-Sen University.

All data are expressed as the mean ± standard deviation. Paired Student’s t-test was used for comparisons between two groups and one-way analysis of variance (ANOVA) was performed for comparisons between more than two groups. P < 0.05 was considered to indicate a statistically significant result. All statistical analyses were conducted by SPSS version 18.0 software (SPSS, Inc.).

## Results

### Chemotherapeutic drug ADM could induce autophagy as well as apoptosis in leukemia cells

**Fig. 1:**
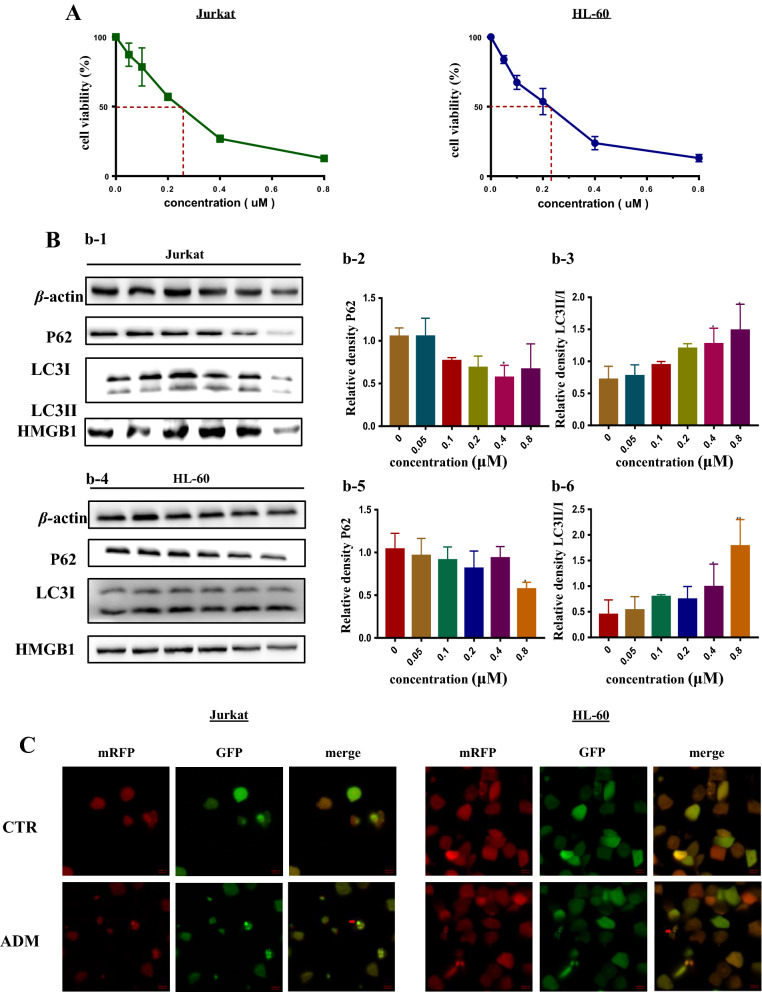

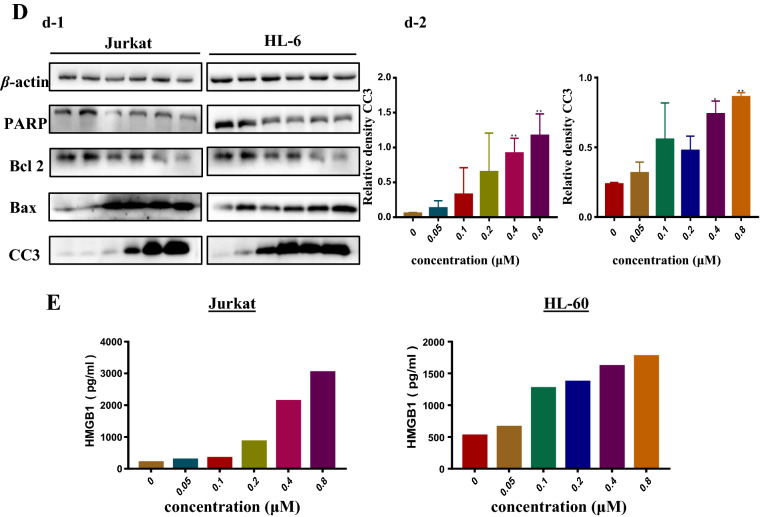
Chemotherapeutic drug ADM induced autophagy and apoptosis in acute leukemia cells. **A** ADM damaged both Jurkat and HL-60 cells dose-dependently. Cell Counting Kit-8 was used to assess the Cell viability. **B** Western blotting was used to detect the expression of LC3II/I and P62 in which β-actin was loading control. Both (b2-b3) and (b5-b6) diagrams are the quantitative data of LC3II/I and P62 in Jurkat and HL-60 cells. **C** Both Jurkat and HL-60 cells that stably expressed mRFP-EGFP-LC3 fusion protein were co-cultured with ADM for 24 h. Confocal microscopic analysis is shown with 630× magnification. Bar = 10 μm, autophagic lysosomes are shown at the red arrow. **D** Western blotting was used to detect the expression of total PAPR, Bcl 2, Bax and cleaved caspase 3 to analyze the level of apoptosis. (d1–d2) Were the diagrams of the quantitative data of cleaved caspase 3 in both leukemia cells. **E** HMGB1 was released when leukemia cells treated with ADM. HMGB1 Elisa kit was applied to assess the supernatant of different group. Data are the mean ± standard deviation of three independent experiments. *P < 0.05, **P < 0.01, compared with the untreated group. *ADM*, Adriamycin

Adriamycin (ADM), also known as Doxorubicin, belongs to the family of anthracycline anticancer drugs, is the cornerstone of the chemotherapy of childhood acute leukemia (AL). As shown in Fig. 1A, both Jurkat and HL-60 cells were damaged by ADM in a dose-dependent manner. Western blotting also applied to analyze ADM’s function on leukemia cells. Figure 1B depicted the upregulation of autophagy in both leukemia cells via western blotting analysis, reflected in the increased ratio of LC3II/I and the continuous decrease of P62, one of the substrates of autophagy. It can be seen in Fig. 1C that both green and red puncta of LC3 were increased in ADM-treated group, which implied that the autophagy flux was augmented as the appliance of ADM in leukemia cells. What’s more, we analyzed the changed of apoptosis when cells treated with ADM by western blotting. It was shown that the level of PARP, one of the substrates and initiates of apoptosis, as well as the ratio of Bcl 2/Bax were gradually decreased. In the meantime, the level of cleaved caspase 3 was increased, which indicated that apoptosis level was elevated when two cell lines treated with ADM (Fig. 1D). Above all, Fig. 1 demonstrates that autophagy and apoptosis are provoked by ADM in the leukemia cells.

### HMGB1 was upregulated and released when leukemia cells treated with ADM

As a damage-associated molecular pattern (DAMP) molecule, HMGB1 was stated that its expression was upregulated in various kinds of cancers. In an attempt to characterize the role HMGB1 plays in acute leukemia, western blotting and HMGB1 Elisa kit were employed to analyze the expression of HMGB1. As presented in Fig. 1B, the expression of HMGB1 was slightly upregulated in lower dose of ADM, however, in higher dose of ADM, the expression of HMGB1 was decreased. The result of Elisa illuminated that HMGB1 was released in a dose-dependent manner (Fig. 1E). To sum up, HMGB1 was upregulated and released under the treatment of ADM, while how the released HMGB1 acts on other AL cells remains unknown.

### Extracellular HMGB1 promoted tumor cells survival by enhancing autophagy and diminishing apoptosis

**Fig. 2:**
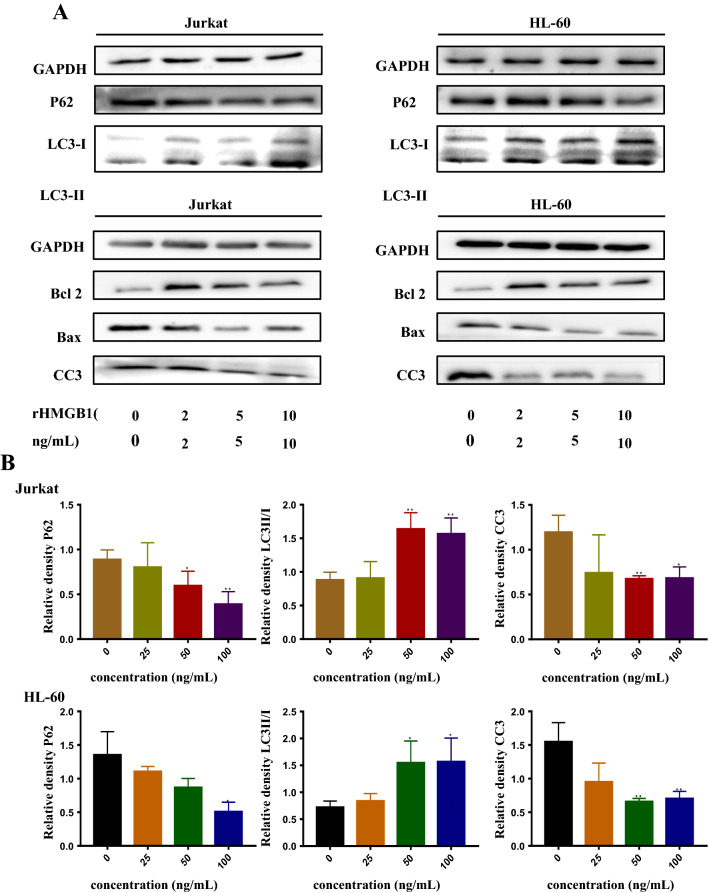
Extracellular HMGB1 induced autophagy and diminished apoptosis in acute leukemia cells. **A** Extracellular HMGB1 protected leukemia cells from the damage of ADM by promoting autophagy and diminishing apoptosis, rHMGB1 (0, 25, 50, 100 ng/mL) was added into leukemia cells before the appliance of ADM (0.4 μM). **B** The diagrams below were the quantitative data of LC3II/I and P62 as well as cleaved caspase 3 in Jurkat and HL-60 cells. GAPDH was used as loading control. Data are the mean ± standard deviation of three independent experiments. *P < 0.05, **P < 0.01, ***P < 0.001 compared with the untreated group

To figure out extracellular HMGB1’s function on leukemia cells, we incubated the cells with extracellular HMGB1 for 24 h before treated with ADM. The changes of autophagy and apoptosis were analyzed by western blotting. On one hand, it can be seen from Fig. 2 that P62 was continuously decreased and the ratio of LC3II/I was increased, which indicated that leukemia cells pretreated with rHMGB1 had a higher level of autophagy than those untreated with rHMGB1. On the other hand, apoptosis was restricted when pretreated with rHMGB1, which was embodied in the descending expression of cleaved caspase 3 as well as the ascending ratio of Bcl 2/Bax and the increase of total Parp. To sum up, extracellular HMGB1 do protect cells from chemotherapeutic drug, which results from its regulations on autophagy and apoptosis in leukemia cells.

### Inhibited the expression of RAGE prevented autophagy induced by extracellular HMGB1

**Fig. 3:**
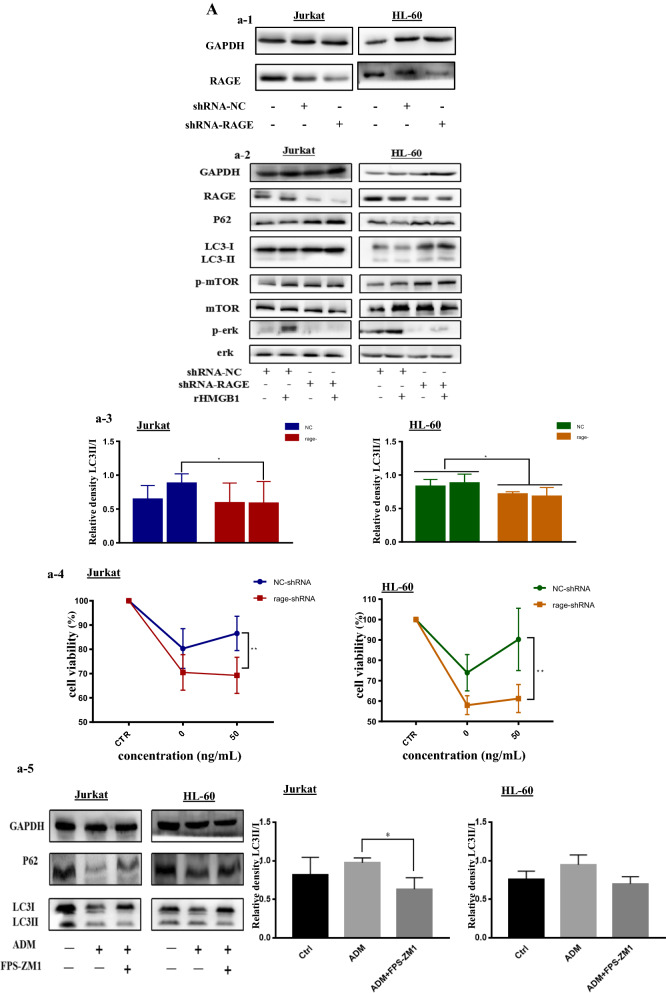

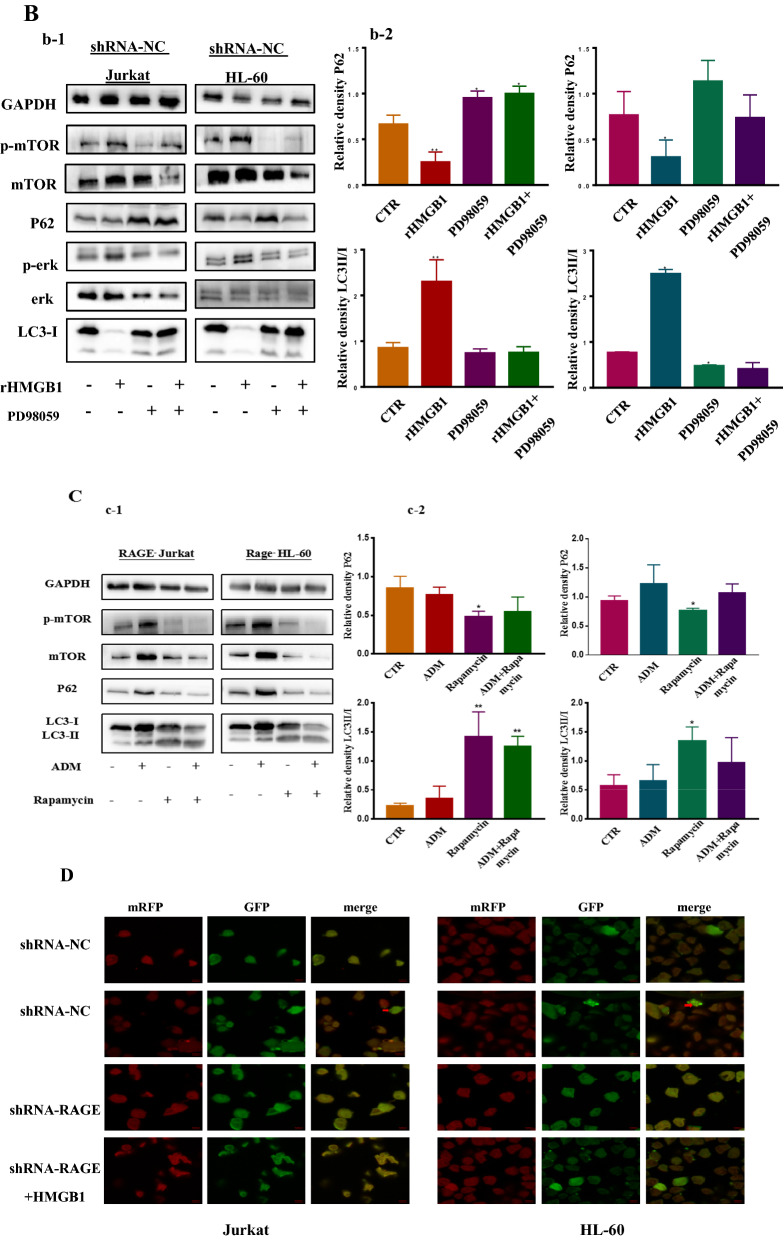
Targeted knocking down of RAGE restricted autophagy which was associated with the phosphorylation of erk and mTOR. **A** (a-1) Both Jurkat and HL-60 cells was transfected with shRNA-RAGE and shRNA-NC for 2 weeks. Then cells were subjected to western blotting to verify the knock down of RAGE protein. (a-2) Targeted knocking down of RAGE decreased autophagy that extracellular HMGB1 induced in leukemia cells. Cells that stably transfected with shRNA-NC and shRNA-RAGE were pretreated with 50 ng/mL rHMGB1 for 24 h, then cells were co-cultured with 0.4 μM ADM for another 24 h. Western blotting was used to detect the alternation of autophagy. (a-3) Was the diagram of the quantitative data of LC3II/I in Jurkat and HL-60 cells respectively. (a-4) Both leukemia cells were pretreated with or without rHMGB1 for 24 h, then ADM (0.4 μM) was applied. Cell Counting Kit-8 was used to assess the cell viability. (a-5) Leukemia cells were treated with ADM (0.4 μM) with or without FPS-ZM1 for 24 h, Western blotting was used to detect the alternation of autophagy. **B** (b-1) Leukemia cells that transfected with shRNA-NC were treated with a small inhibitor of ERK2/1, 10 μM PD98059 together with 50 ng/mL rHMGB1 for 12 h, then 0.4 μM ADM was added into cells. Western blotting was used to detect the change of autophagy and the phosphorylation of ERK and mTOR. (b-2) Showed the quantitative data of P62 and LC3II/I of leukemia cells. **C** Cells that stably transfected with shRNA-RAGE were pretreated with the small inhibitor of mTOR, Rapamycin (100 nM) for 6 h, following the treatment of ADM (0.4 μM). Cells were subjected to western blotting to assess the level of autophagy and the phosphorylation of mTOR. (c-2) Presented the quantitative data of P62 and LC3II/I of leukemia cells. GAPDH was used as loading control. **D** Both Jurkat and HL-60 cells that transfected with shRNA-NC and shRNA-RAGE were transfected with mRFP-EGFP-LC3 virus. In the subsequent experiment, cells were pretreated with 50 ng/mL rHMGB1 for 24 h and then 0.4 μM ADM was added for another 24 h. Confocal microscopic analysis is shown with 630× magnification. Data are the mean ± standard deviation of three independent experiments. *P < 0.05, **P < 0.01, compared with the untreated group

**Fig. 4:**
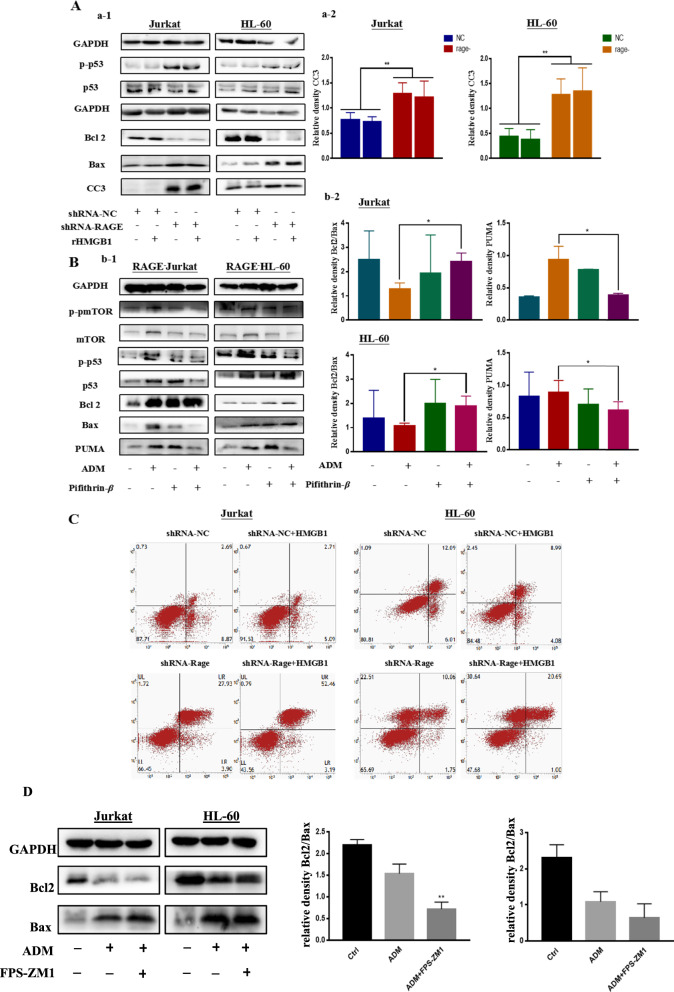
The ablation of RAGE enhanced apoptosis in leukemia cells and the apoptosis was related with the phosphorylated of P53. **A** (a-1) Cells that stably transfected with NC shRNA and RAGE shRNA were pretreated with 50 ng/mL rHMGB1 for 24 h, then cells were co-cultured with 0.4 μM ADM for another 24 h. Western blotting was used to detect the alternation of apoptosis. (a-2) Was the quantitative data of cleaved caspase 3/GAPDH. **B** (b-1) Cells that stably transfected with shRNA-RAGE were pretreated with the small inhibitor of p53, Pifithrin-β (1 μM) for 12 h, following the treatment of ADM (0.4 μM). Cells were subjected to western blotting to assess the level of apoptosis and the phosphorylation of P53. (b-2) Were the quantitative data of Bcl 2/Bax and PUMA/GAPDH in both leukemia cells. **C** Cells in step A were collected and washed with PBS for 2 times. Annexin V APC-A and APC-Cy7-A were used to evaluate the level of apoptosis in different groups. **D** Leukemia cells were treated with ADM (0.8 μM) with or without FPS-ZM1 for 24 h, Western blotting was used to detect the alternation of apoptosis. Data are the mean ± standard deviation of three independent experiments. *P < 0.05, **P < 0.01, compared with the ADM-treated NC group

**Fig. 5:**
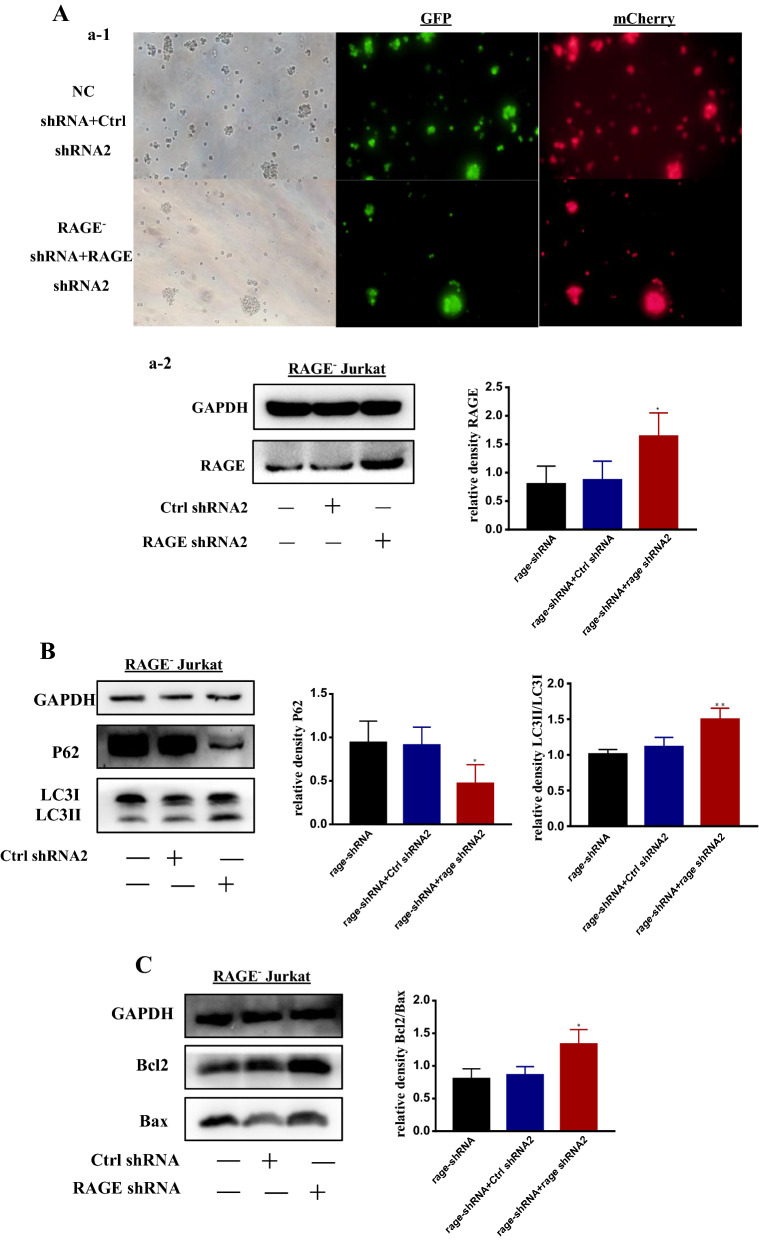
Re-expression of RAGE with RAGE shRNA restored the level of autophagy and apoptosis induced by ADM in RAGE^−^ Jurkat cells. **A** (a-1) RAGE was overexpressed in RAGE-knockdown Jurkat cells. RAGE^−^ Jurkat cells were transfected with shRNA-RAGE and shRNA-NC for 2 weeks. Microscopic analysis is shown with 200× magnification. Then cells were subjected to western blotting to verify the knock down of RAGE protein, as shown in a-2. **B** The transfected cells were treated with ADM (0.4 μM). Western blotting was used to detect the alternation of autophagy. The diagrams of the quantitative data of LC3II/I and P62 in transfected cells were presented. **C** Cells that stably transfected with shRNA2-RAGE were treated with ADM (0.8 μM),western blotting was used to assess the level of apoptosis. Data are the mean ± standard deviation of three independent experiments. *P < 0.05, **P < 0.01, compared with the RAGE^−^ Jurkat group

To characterize the role RAGE plays in leukemia, we inhibited the expression of RAGE in two leukemia cell lines by lentivirus infection and FPS-ZM1, a small molecular inhibitor of RAGE. Western blotting was used to analyze the expression of RAGE (Fig. 3A a-1). As shown in Fig. 3A a-4, leukemia cells were damaged by ADM while RAGE^−^ cells were even worse and could not be protected by rHMGB1. Then experiments had been conducted to examined RAGE’s role in HMGB1 induced autophagy. We can see that FPS-ZM1 restrained autophagy induced by ADM, as shown in Fig. 3A a-5, the degradation of P62 was curbed and the ration of LC3II/I was decreased. Detection of LC3II/I by immunoblotting or immunofluorescence delineated that knock down of RAGE curbed the autophagy and prevent the autophagy induced by HMGB1 (Fig. 3A a-2, D). The limited degradation of P62 also showed that autophagy was prevented (Fig. 3A a-2). There was a significant difference between NC and RAGE^−^ group, which demonstrated that RAGE is crucial in the progression of autophagy.

### HMGB1/RAGE induced autophagy was associated with increased level of phosphorylated ERK2/1 and decreased level of phosphorylated mTOR

Autophagy is initiated by many mechanism, in which PI3K/AKT/mTOR and MEK/ERK2/1 are crucial and frequently studied. To gain insight in the molecular mechanism of HMGB1/RAGE induced autophagy, we compared the difference of the level of phosphorylated ERK and phosphorylated mTOR between shRNA-NC and shRNA-RAGE cells. From Fig. 3A a-2, we can see that the level of phosphorylated ERK was declined while the level of phosphorylated mTOR was increased. To further research the relationship between erk, mTOR and autophagy, we used small molecule inhibitor of ERK (PD98059) and mTOR (Rapamycin) to curb the activation of ERK and mTOR. It can be seen from Fig. 3B that the level of phosphorylation of ERK was decreased and autophagy was limited accordingly, which was ascertained by decreasing P62 and increasing ratio of LC3II/I in both RAGE knocked down tumor cells. Unexpectedly, phosphorylated mTOR was declined. When the shRNA-RAGE cells were treated with Rapamycin, autophagy was upregulated as the phosphorylated mTOR declined (Fig. 3C c-1).

### Inhibited the expression of RAGE increased apoptosis in leukemia cells and the apoptosis was related with the phosphorylated of P53

Apoptosis, also known as programmed cell death, is one of the best characterized form of cell-death process. As mentioned above, apoptosis was halted when employed extracellular HMGB1 into leukemia cells. To identify the function of RAGE in the progression of apoptosis, firstly we knocked down RAGE and we can clearly see that the ascending level of cleaved caspase3, which indicates the upregulation of apoptosis in leukemia cells following chemical treatments. Meanwhile, FPS-ZM1 enhanced the apoptosis induced by ADM in both leukemia cells, as shown in Fig. 4D, the descending ratio of Bcl2/Bax. Furthermore, extracellular HMGB1 had less protective impact or even became harmful on cells without RAGE (Fig. 4A, C). We found that depletion of RAGE increased phosphorylation of p53 (Fig. 4A). P53 is the one of tumor suppressor and is a major checkpoint of apoptosis. Activating P53 can initiate apoptosis when cells encounter intracellular genotoxic stress. We also found that the level of pro-apoptotic bcl2-family members, Bax and PUMA in RAGE knocked down leukemia cells was higher than control group, while the level of anti-apoptosis protein Bcl2 was reduced. To clarify the role that P53 plays in apoptosis results from knocking down of RAGE. We use pifithrin *β* (PFT-*β*), a specific antagonist of p53, to inhibit the phosphorylation of p53 in RAGE^−^ leukemia cells. It can be seen from Fig. 4B that inactivate P53 increased the ratio of Bcl 2/Bax while downregulate the expression of PUMA following chemotherapy treatments. What’s more, the phosphorylation of mTOR also decreased. Therefore, it is suggested that RAGE could limit apoptosis and this process was associated with the phosphorylation of P53.

### Re-expression of RAGE with RAGE shRNA restored the level of autophagy and apoptosis induced by ADM in RAGE^−^ Jurkat cells

To confirm that the expression of RAGE was crucial in the regulation of autophagy and apoptosis, we examined the re-expression of RAGE through lentivirus transfection. As shown in Fig. 5A, western blotting was used to detect the expression of RAGE. Re-expression of RAGE in the knockdown Jurkat cells restored autophagy and apoptosis induced by ADM to basal levels. Figure 5B depicted that the re-expression of RAGE increased the degradation of P62 and increased ratio of LC3II/LC3I, which indicated the upregulation of autophagy. Figure 5C illuminated that the re-expression of RAGE increased the ratio of Bcl2/Bax, which revealed that the Bcl2 related apoptosis was declined. These findings support a critical role for RAGE in the regulation of autophagy and apoptosis induced by ADM in leukemia cells.

### HMGB1/RAGE induced the expression of drug resistance protein by activating NF-κB pathway

**Fig. 6:**
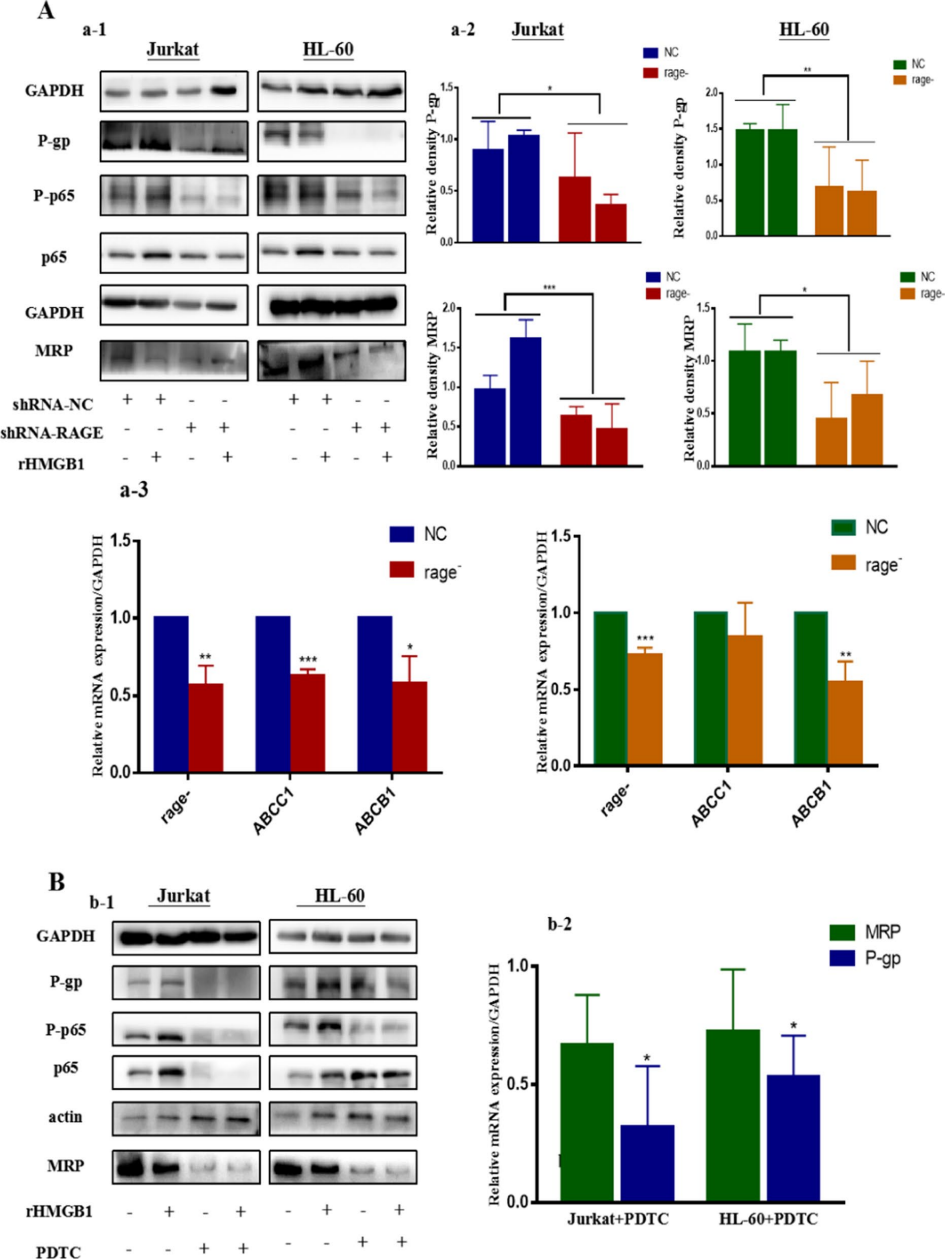
HMGB1/RAGE induced the expression of drug resistance protein via NF-kB pathway. **A** (a-1) Leukemia cells that stably transfected with NC shRNA and RAGE shRNA were pretreated with 50 ng/mL rHMGB1 for 24 h, then cells were co-cultured with 0.4 μM ADM for another 24 h. Western blotting was used to detect the expression of P-gp and MRP as well as the phosphorylation of P65. (a-2) Was the quantitative data of P-gp and /GAPDH in both leukemia cell lines. (a-3) Was the quantitative data of RAGE, ABCB1 and ABCC1/GAPDH in mRNA expression. **B** (b-1) Cells that stably transfected with shRNA-NC were pretreated with the small inhibitor of NF-κB, PDTC (1 μM) for 12 h, following the treatment of ADM (0.4 μM). Cells were subjected to western blotting to assess the expression of P-gp and MRP and the phosphorylation of P65. (b-2) Were the mRNA expression of P-gp and MRP of cells treated with PDTC compared to NC group. GAPDH was used as loading control. Data are the mean ± standard deviation of three independent experiments. *P < 0.05, **P < 0.01

Over-expression of drug resistance protein such as P-gp and MRP is one of the reasons of existence of multi-drug resistance (MDR) and obstacles of anti-cancer treatments. From Fig. 6A we can see that extracellular HMGB1 actually could upregulate the expression of drug resistance protein, and this process was also RAGE-dependent. In the meantime the activation of NF-κB was halted due to the depletion of RAGE. In the subsequent experiments, PDTC, one of the antagonist of NF-κB, was applied before chemotherapy. Western blotting and qPCR were used to assess the expression of P-gp and MRP. Figure 6B showed that the expression of P-gp and MRP was sharply decreased as the inactivation of NF-κB. It is implied that HMGB1/RAGE can provoke the expression of P-gp and MRP primarily by the activation of NF-κB.

### Blocking HMGB1/RAGE axis increased sensitivity to chemotherapy in vivo

**Fig. 7:**
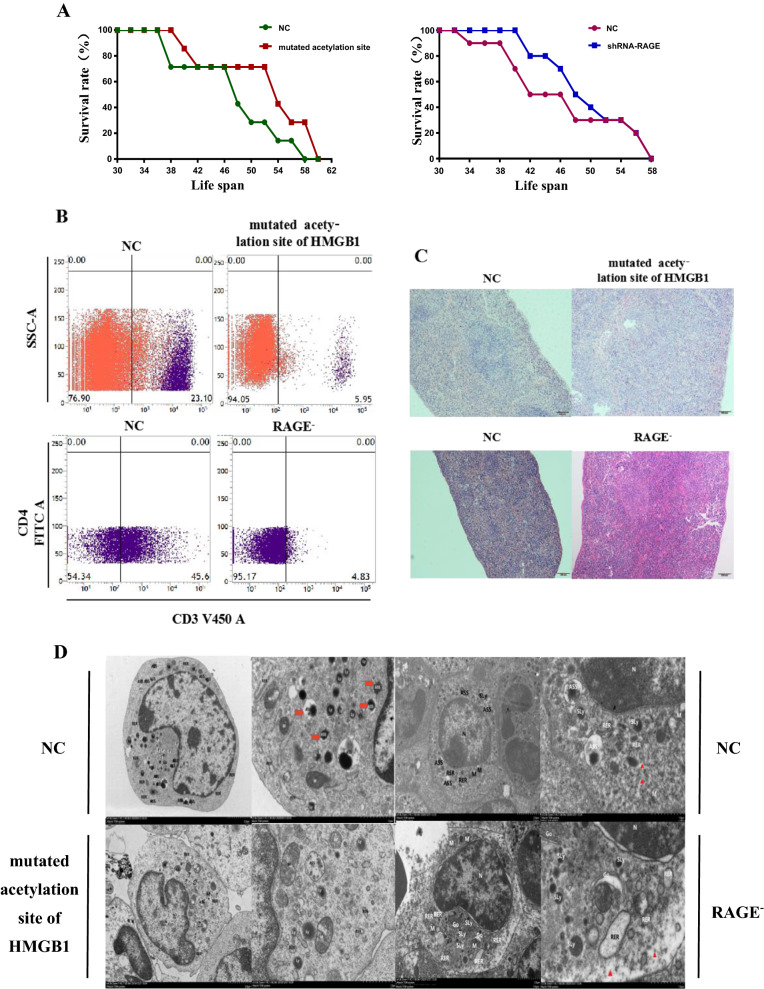
The obstruction of HMGB1/RAGE increased sensitivity to chemotherapy in vivo. **A** 5 × 10^6^ Jurkat cells with stable transfection of control and Rage shRNA as well as mutated acetylation site of HMGB1 were injected into NOD/SCID mouse via intravenous injection to establish a model of leukemia mouse. Low dosage of ADM (1 mg/per mouse) was injected into mouse every other day. The diagram showed us the life span of mouse in different groups. **B** Bone marrow sediment of mouse was removed and stained with BV421-CD3 before flow cytometry to detect the proportion of leukemia cells in bone marrow. **C** When mouse come to the endpoint of death, spleen was separated and fixed with 4% paraformaldehyde. Pathological section was made by using hematoxylin–eosin staining. Microscopic analysis is shown with 40× magnification. **D** Bone marrow sediment of mouse was removed and subjected to transmission electron microscopy to observe autophagosome-like structures (indicated by red triangle)

To figure out whether knock-down of RAGE increases sensitivity towards chemotherapy in vivo, we established a model of T-ALL mouse. Figure 7A depicted that mouse injected with shRNA-RAGE or shRNA-mutated acetylation site had a better life span than those injected with shRNA-NC cells. From Fig. 7B, C we can see that mouse that injected with shRNA-RAGE or shRNA-mutated acetylation site had less burden of leukemia cells in spleen and marrow. As shown in Fig. 7D, transmission electron microscopy was employed to calculate the number of autophagosome-like structures and it was suggested that mouse that injected with shRNA-RAGE or shRNA-mutated acetylation site had a lower level of autophagy than those with shRNA-NC cells. From above mentioned, it is suggested that the interference of HMGB1/RAGE can alleviate the leukemia burden in vivo.

## Discussion

Currently, drug resistance remains the main obstacle to overcome when treating acute leukemia with chemotherapy treatments, which is also the primary reason for the development of refractory and recurrent leukemia [6, 40]. A great number of studies had focused on this aspect, in which the elevation of autophagy and the over-expression of multiple drug-resistant protein as well as the suppression of apoptosis were demonstrated to contribute greatly to the occurrence of drug resistance. ADM is one of the cornerstone chemotherapy drugs for leukemia, and the cytotoxicity mediated by ADM is drug-induced DNA damage [40]. High-mobility group protein 1 (HMGB1), known as one of the most characterized damage associated molecular pattern molecule (DAMP), could be passively released when cells are under stress or injured, leading to the regulation of autophagy, apoptosis as well as immunity and so on [41]. Han et al. demonstrated that ADM could trigger the translocation and release of HMGB1 in tumor cells, which induced cytoprotective autophagy in K562 cells by activating the MEK/ERK2/1 signalling pathway [42]. Kong et al. revealed that HMGB1 promoted the dissociation of Beclin1–Bcl-2 complexes and modified Beclin1 binding to PI3k catalytic subunit 3, thus initiating autophagosome formation [24]. Liu et al. showed that extracellular HMGB1 can promote autophagy and prevent necroptosis in acute myeloid leukemia cells [17]. In this study, as shown in Fig. 1, ADM do upregulate autophagy and apoptosis in a dose-dependent manner accompanied by the release of HMGB1 in acute leukemia cell, which was in accordance with the studies mentioned above. However, the overall expression of HMGB1 was not significantly changed, at the higher dosage of ADM, the expression of HMGB1 was sharply decreased. Combined with the result of Elisa, we assumed that leukemia cells might encounter necroptosis and HMGB1 was released rapidly. As presented in Fig. 2, we pretreated leukemia cells with rHMGB1 before treated with ADM and found that extracellular promoted autophagy and protected cells from apoptosis, which suggested that extracellular HMGB1 play an protective role in chemotherapeutic treatments. However, how the released HMGB1 acts on leukemia cells remain elusive.

The receptor for advanced glycation end products (RAGE) is the first demonstrated binding partner to HMGB1 [43]. RAGE is a multiligand receptor of the immunoglobulin superfamily in cell surface molecules, acting as counter-receptor for diverse molecules such as AGEs, S100 proteins, HMGB1 and so on [28]. RAGE and its ligands are over-expressed in multiple cancers [33, 44]. What’s more, it was reported that HMGB1-RAGE signalling triggered activation of several key cell signalling pathways, such as MAPK, NF-κB, and Rac/Cdc42 in nasopharyngeal carcinoma [45]. Study had also found that in a murine model, targeted knocking down of RAGE diminished the autophagy and prevented the development of early pancreatic neoplasia possibly through IL-6-induced phosphorylation of STAT3 [46]. In pancreatic cancer, it was reported that HMGB1 or its receptor RAGE by RNAi or antisense nucleotide could inhibit cell invasion and augment chemotherapy sensitivity partly by down-regulation of autophagy [47]. Therefore, we hypothesized that extracellular HMGB1 acts on leukemia cells mainly by interacting with RAGE. In present study, by targeting knocking down RAGE, we found that the level of autophagy was sharply decreased and the apoptosis was increased in both leukemia cell lines (Fig. 3). What’s more, the absence of RAGE halted the protection of rHMGB1. Leukemia cells was aggravated even worse with HMGB1 due to the ablation of RAGE. On one hand, studies found that extracellular HMGB1 can interact with other receptors such as TLR4 and CXCR4 and then worsen inflammation. On the other hand, the arising ROS level can oxidize HMGB1, which makes it more liable to bind to TLR4 [23, 26], and then leads to the deterioration of leukemia cells.

**Fig. 8:**
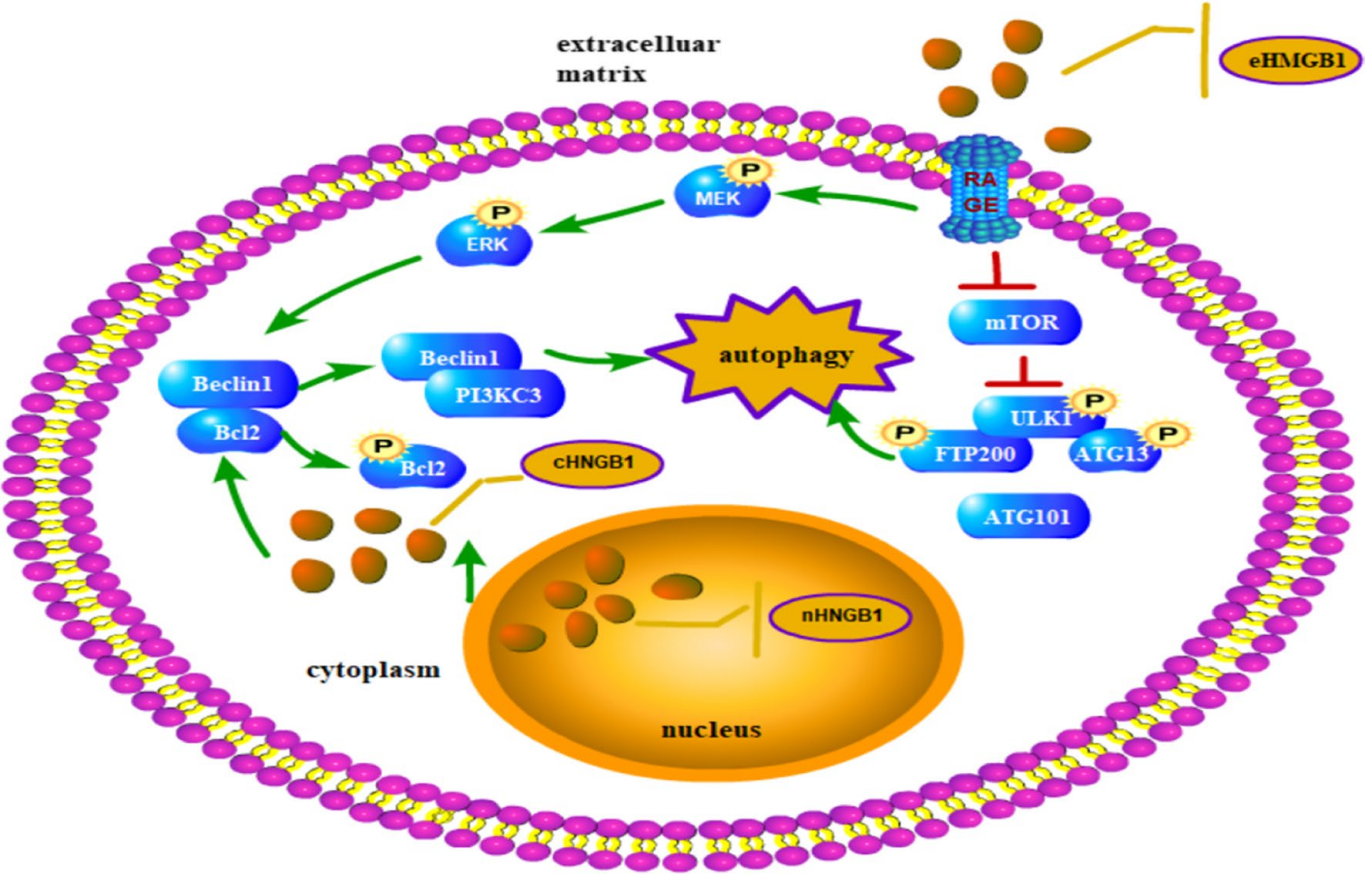
Schematic representation of the potential autophagy-related signaling pathway in leukaemia. Under stress condition, HMGB1 translocates to the cytoplasm and then release into the extracellular matrix. Both cHMGB1 and eHMGB1 dislocate the Beclin 1–Bcl 2 complex, facilitating the formation of PI3K3C-Beclin 1, which leads to the initiation of autophagy. Besides, extracellular HMGB1 can suppress the activation of mTOR and then phosphorylate the ULK1-ATG13-FIP200 complex and then promote autophagy

As shown in Fig. 8, MAPK/ERK and mTOR are crucial in the initial of autophagy. Pan et al. recently reported that HMGB1 enhanced autophagy through activating the MEK/ERK1/2 signalling pathway and promote docetaxel resistance in human lung adenocarcinoma [48]. Li et al. postulated that HMGB1 facilitates the growth and advancement of clear cell renal cell carcinoma via ERK1/2 stimulation, which is partly mediated by RAGE [49]. While Liu et al. reported that HMGB1 enhanced autophagy and chemotherapy resistance through MAPK/mTOR signalling pathway in K562 cells [50]. In present study, we make an attempt to figure out the relationship between ERK and mTOR in autophagy induced by HMGB1/RAGE. We found that in RAGE^−^ cells, the phosphorylation of ERK was decreased and by contrast, the phosphorylation of mTOR was elevated, along with the restriction of autophagy. By inhibiting the phosphorylation of ERK by PD98059, a small molecular inhibitor of MEK/ERK, autophagy was strikingly decreased in leukemia cells. To our surprise, the phosphorylation of mTOR was repressed. Then Rapamycin was applied to the RAGE^−^ cells, autophagy was upregulated conversely as the phosphorylation of mTOR was reduced.

It was believed that Beclin-1 is crucial in the autophagy initiated by the activation of mTOR, while the dislocation of Beclin-1 and Bcl 2 resulted from the activation of MEK/ERK. And it is suggested that the absence of RAGE lead to down-regulation of autophagy via the increased phosphorylation of mTOR and the decreased phosphorylation of ERK, in which MEK/ERK is supposed to be the upstream of the mTOR signal. The activation of ERK is more crucial in the autophagy induced by HMGB1/RAGE.

P53, one of the most studied tumor suppressor genes, had been found frequently mutated in leukemia cells and assumed to have a cross link to HMGB1 to regulate autophagy and apoptosis in the setting of cell stress [51]. What’s more, P53 induced apoptosis, known as intrinsic apoptosis, is mainly by activating the expression of pro-apoptotic protein such as PUMA and Bax, along with the decline of anti-apoptotic protein such as Bcl-2 and MCl-1 [13, 52]. HMGB1/RAGE also have great association with apoptosis. It is found that HMGB1-mediated autophagy decreases vincristine-induced apoptosis in gastric cancer partly via upregulation of Mcl-1, a Bcl-2 family member [53]. In present study, we found that targeted knocking down RAGE induce apoptosis and prevented the protective effect of extracellular HMGB1 with the change of the Bcl-2 family member. We proposed that HMGB1/RAGE related apoptosis is P53 dependent. The increased phosphorylation of P53 was observed in RAGE^−^ cells. And then we use pifithrin B, a small molecular inhibitor of P53 to inhibit the activation of P53 and we found that it abrogated the enhanced apoptosis due to the ablation of RAGE. Meanwhile, we can see that the decline of PUMA and Bax and the increase of Bcl-2 when treated with pifithrin B (Fig. 4). To our interest, the decrease the level of phosphorylation of mTOR was reduced, which suggested that P53 can be the hub of the regulation of autophagy and apoptosis related with HMGB1/RAGE. The association between P53 and HMGB1/RAGE require further research. And the studies above suggests that HMGB1/RAGE help protect tumor cells from cytotoxic insult partly by limiting a P53-dependent pathway of programmed cell death.

It is established that NF-κB is one of the signaling ways activated by HMGB1/RAGE [16, 54, 55]. And NF-κB is a key protein that affect the progression of tumors. Upon activation, NF-κB translocates to the nucleus and subsequently binds to DNA sequences to stimulate transcription of target genes, ABC family members such as ABCC1 and ABCB1 were included [38, 56, 57]. ABCC1 and ABCB1 transporters conferred resistance to multidrug resistance (MDR), an intrinsic or acquired cross-resistance toward different chemotherapeutic drugs, which has been considered as one of the primary reasons for the failure in cancer chemotherapy. Meanwhile, numberous studies demonstrated that anthracyclines such as doxorubicin and daunorubicin were substrates of MRP1 and P-gp, encoded by ABCC1 and ABCB1 [34, 58]. However, the relationship between HMGB1/RAGE and ABC transporters are unclear. In present study, the phosphorylation of NF-κB as well as the expression of P-gp and MRP was decreased following the absence of HMGB1 and RAGE. When used a small molecular inhibitor PDTC to prevent the phosphorylation of NF-κB, the expression of P-gp and MRP were decreased both in mRNA and protein level. It is indicated that HMGB1/RAGE might induce NF-κB to translocate into nucleus and promoting the expression of ABCB1 and ABCC1.

Acetylation of HMGB1 is crucial in the translocation and release of HMGB1 [22]. Mice that injected with mutated HMGB1 cells or RAGE^−^ cells had better life quality and less leukemia burden. What’ s more, the result of transmission microscope validated the important role HMGB1/RAGE plays in autophagy. It is suggested that HMGB1, as a DAMP molecule, is upregulated and released to adapt leukemia cells to chemotherapy, while RAGE, acting as HMGB1’s receptor, affects downstream signalling and promotes leukemia cell survival by regulations of autophagy, apoptosis as well as multidrug resistant proteins. To sum up, HMGB1/RAGE shape the accommodating micro-environment for leukemia cells to prevent the harm of chemotherapy.

In conclusion, the present study demonstrates the role HMGB1/RAGE axis plays in promoting acute leukemia cells survival by diminishing apoptosis and increasing autophagy and the expression of multiple drug resistant protein. These data support the development of novel therapy targeting HMGB1/RAGE. Interfering with HMGB1/RAGE can be a potential means to cure acute leukemia.

## Data Availability

The datasets used and/or analysed during the present are available from the corresponding author on reasonable request.

## Abbreviations

HMGB1: High mobility group box 1
ADM: Adriamycin
RAGE: Receptor for advanced glycation end products
DAMP: Damage-associated molecular pattern
MAPK: Mitogen activated protein kinases
ERK: Extra cellular signal regulated kinases
AMPK: (AMP)-activated protein kinase
P-gp: P-glycoprotein
MRP: ATP-binding cassette transporter
ABC: ATP-binding cassette transporter
NF-κB: Nuclear factor κB
LC3: Microtubule—associated protein 1 light chain 3
P62: Sequestosome 1
PI3K: Phosphatidylinositol-3-Kinase
mTOR: Mammalian Target of Rapamycin
